# Monitoring Non-dipping Heart Rate by Consumer-Grade Wrist-Worn Devices: An Avenue for Cardiovascular Risk Assessment in Hypertension

**DOI:** 10.3389/fcvm.2021.711417

**Published:** 2021-07-23

**Authors:** Tomas Baka, Fedor Simko

**Affiliations:** ^1^Institute of Pathophysiology, Faculty of Medicine, Comenius University, Bratislava, Slovakia; ^2^3rd Department of Internal Medicine, Faculty of Medicine, Comenius University, Bratislava, Slovakia; ^3^Institute of Experimental Endocrinology, Biomedical Research Center, Slovak Academy of Sciences, Bratislava, Slovakia

**Keywords:** hypertension, heart rate, non-dipping heart rate, wearables, smartwatch, wrist-worn device

## Introduction

Elevated resting heart rate (HR) is a recognized cardiovascular risk factor in populations with or without cardiovascular affliction (1, 2). High HR comes with increased myocardial oxygen demand in a decreased diastolic filling time and coronary perfusion potentially interfering with the myocardial oxygen demand/supply balance and cardiac performance. In addition, elevated HR may underlie the abnormal vascular shear and pulsatile stress associated with endothelial dysfunction, vascular stiffness and oxidative stress (3, 4). Similarly to blood pressure (BP), HR undergoes circadian variations primarily dependent on the sympathovagal balance. Non-dipping, i.e., blunted BP or HR decline overnight (by <10% of the daytime mean), is coupled with an adverse cardiovascular prognosis (5). Although sustained relative sympathetic over-activation at night is considered to be pivotal in the pathophysiology of non-dipping HR, other factors may also participate, such as the disrupted endogenous circadian rhythmicity of the central clock in the suprachiasmatic nucleus, the cardiac clock in the sinoatrial node, or circulating neurohumoral factors, including catecholamines and glucocorticoids (6–8). Non-dipping HR is rather neglected during cardiovascular risk assessment and is thus omitted from risk management strategies (5). Nevertheless, a restricted number of studies have shown that non-dipping HR may be of adverse prognostic value in the general population (9) and various cardiovascular pathologies, including hypertension (10), chronic kidney disease (11) and type 2 diabetes (12). Indeed, a 10% reduction of overnight HR decline in a general and hypertensive population was associated with an increased risk of all-cause mortality by 34 and 30%, respectively (9, 10). Of note, the overnight HR dip was shown to be progressively attenuated by physiological ageing (9, 13, 14). Thus, age should be taken into account in cardiovascular risk assessment using non-dipping HR to yield valid results. In fact, non-dipping HR was found to be an independent risk factor of adverse cardiovascular outcomes after adjustment for several covariates, such as age, gender, blood pressure, body mass index, co-morbidities, or treatments (9, 10, 13, 14). For instance, Ben-Dov et al. (9) calculated that the risk of non-dipping HR increased by 13% per 5 years of age and the risk of all-cause mortality categorically increased by 45% with non-dipping HR after adjustment for multiple covariates including age. Unfortunately, research into non-dipping HR is not advancing at a desirable pace; thus, only seldom has data on this topic emerged in recent decades.

## Consumer-Grade Wrist-Worn Devices for Monitoring Non-Dipping HR in Hypertension

Consumer-grade wearable devices capable of monitoring health represent an ever-growing market that is expected to reach 27.5 billion USD by 2026. Wrist-worn devices, including smartwatches and fitness trackers, account for more than half of all wearable devices being used (15). Various sensors and technologies are utilized by these devices to gather health parameters, but photoplethysmography (PPG) is considered quintessential. PPG is an optical measurement technique to detect blood volume changes in subcutaneous tissue based on the interaction of light (absorption or reflection) with blood in the tissue, thus allowing for a beat-to-beat pulse signal interpretation. The PPG-derived waveform is used to extrapolate several parameters, including HR, heart rhythm, and indices of autonomic balance (16). The identification of motion and noise artifacts by advanced artificial intelligence, particularly machine learning, has significantly improved the accuracy of HR measurement at rest, and even during physical activity, or arrhythmia (15). A set of clinical studies assessed the accuracy of HR monitoring by consumer-grade wrist-worn devices under varying physical exertion. In general, the accuracy of wrist-worn devices was best at rest (reaching 90–95% agreement with electrocardiogram, ECG), worsened with exercise, and varied with exercise type (17–21). Indeed, a meta-analysis of 44 validation studies comprising 738 effect sizes across 15 consumer-grade wrist-worn devices showed that HR data collected by these devices during common activities under free-living conditions (sleep, rest, treadmill, post-exercise, and daily living activities) closely resembles HR data derived from ECG or chest-strap telemetry. Importantly, the mean differences between wrist-worn devices and a criterion HR measurement (ECG or chest-strap telemetry) were minimal during night-time sleep (−0.40 bpm) and daytime rest (−0.01 bpm) or when walking/running (−0.51 bpm). A sizeable difference was found in only two vigorous training activities: cycling and resistance training (22). Recently, a multiple component algorithm was developed to approximate circadian HR changes using HR and activity and sleep data measured by wrist-worn devices. A validation study comprising 47 individuals using a wrist-worn device showed this algorithm to accurately reproduce a circadian HR pattern (23). Thus, the above data provide evidence that wrist-worn devices are able to measure HR across night-time sleep and common daytime activities and reproduce a circadian HR pattern under free-living conditions with high accuracy.

Interestingly, wrist-worn devices were shown to accurately and reliably determine short-term and 24-hour (24-h) heart rate variability (HRV) by extrapolation from PPG-derived waveforms (24–28). Time- and frequency-domain HRV indices are considered a dynamic metric of the autonomic nervous system, including cardiac autonomic balance or baroreflex sensitivity, thus making HRV a disease marker of clinical conditions with increased cardiovascular risk and a predictor of adverse cardiovascular outcomes (29). Indeed, reduced circadian HRV was shown to be associated with autonomic imbalance (30) and reduced baroreflex sensitivity (31, 32). Thus, considering 24-h HRV together with other metrics simultaneously measured by wrist-worn devices (e.g., 24-h HR, non-dipping HR or activity/sleep data) may further improve cardiovascular risk assessment (33). A cross-sectional study with 8,203,261 subjects from 74 countries showed that (i) diverse metrics of cardiac autonomic health can be derived from 24-h HRV measured by wrist-worn devices; and (ii) the correlation of 24-h HRV with other data obtained by these devices, such as physical activity, is feasible and can potentially be utilized to improve cardiovascular risk assessment (28).

Nonetheless, PPG comes with various sources of noise that may interfere with HR measurement if left unmitigated by post-sensing processing *via* algorithms and data analytics. Such sources of noise can be primarily divided into three categories: (i) individual variation, e.g., skin tone, obesity, age, and gender; (ii) physiology, e.g., respiratory rate, venous pulsation, and local body temperature; and (iii) external factors, e.g., motion artifacts, ambient light, and pressure applied to the skin (34). Of note, in validation studies, wrist-worn devices using PPG for HR measurement achieved high agreement with standard ECG, or chest-strap telemetry, indicative of minimal interference by noise or other artifacts (22).

Importantly, wearable devices are gaining popularity among both patients and physicians, as the use of these devices by consumers nearly quadrupled in a 4-year period (from 9% in 2014 to 33% in 2018) and 2 in 3 healthcare professionals surveyed intend to use wearable devices for remote patient monitoring (15).

**Figure 1:**
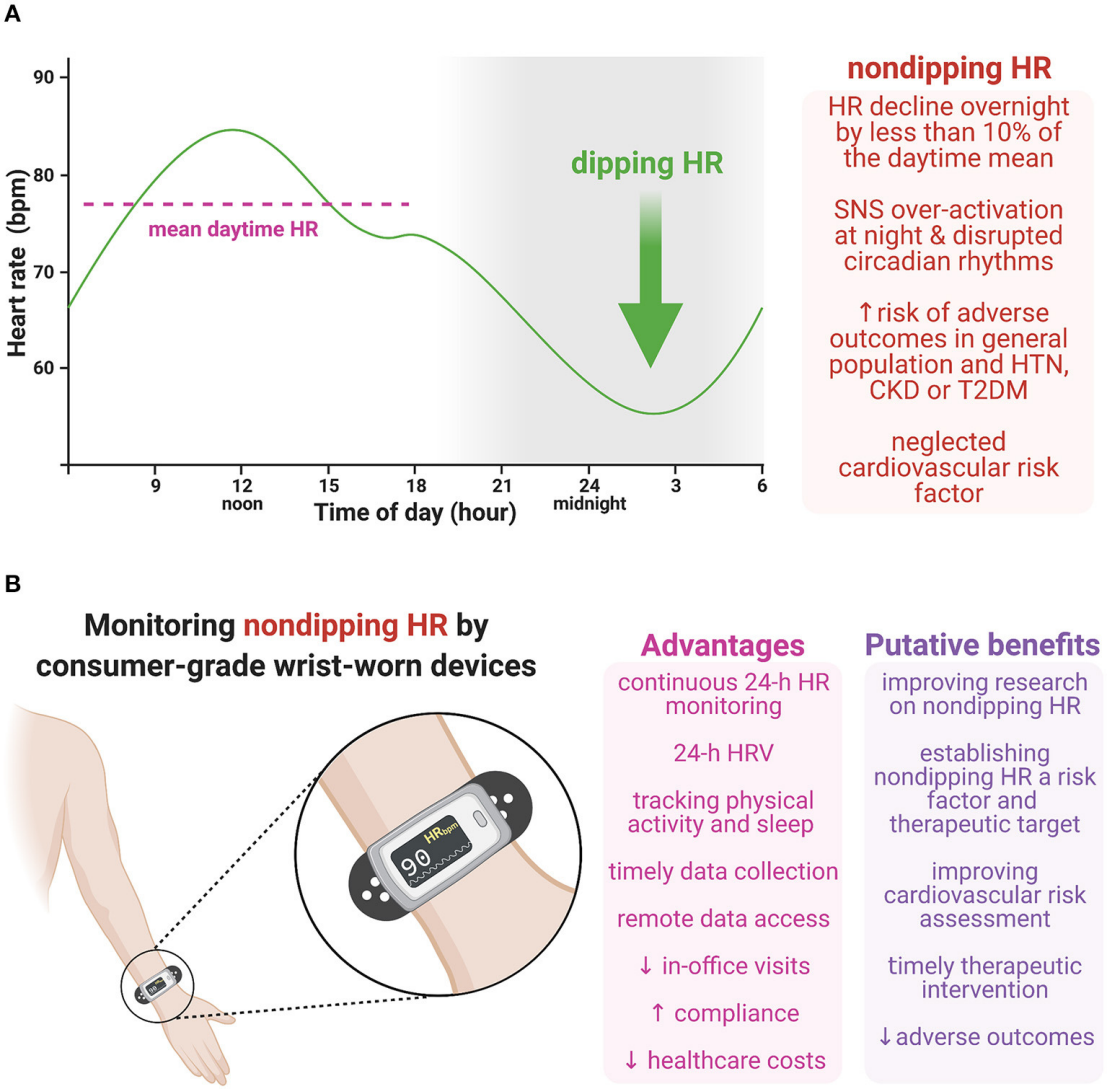
**(A)** Non-dipping heart rate (HR), i.e., HR decline overnight by <10% of the daytime mean, is coupled with an increased risk of adverse outcomes in the general population and various cardiovascular pathologies. Although sustained sympathetic over-activation at night is considered to be pivotal in the pathophysiology of non-dipping HR, other factors may also be involved, such as the disrupted endogenous circadian rhythmicity of the central clock in the suprachiasmatic nucleus, the cardiac clock in the sinoatrial node, or circulating neurohumoral factors, including catecholamines and glucocorticoids. **(B)** Consumer-grade wrist-worn devices provide continuous HR monitoring during a 24-hour (24-h) period (or even longer), with simultaneous physical activity and sleep tracking and determining HR variability (HRV). Moreover, these devices enable timely data collection with no subjective data reporting or secondary data entry, and remote access to data coming with increased patient compliance and reduced in-office visits and healthcare costs. Monitoring non-dipping HR with wearable devices could improve the pathophysiological understanding of this phenomenon, show its prognostic value in cardiovascular as well as other pathologies and help to develop a therapeutic approach targeting non-dipping HR. Moreover, considering 24-h HRV together with other metrics simultaneously measured by wrist-worn devices (e.g., 24-h HR, non-dipping HR or activity/sleep data) might further improve cardiovascular risk assessment. Thus, this cost-effective approach could potentially improve cardiovascular risk assessment, reduce adverse outcomes by timely therapeutic intervention, and curb long-term healthcare expenditures. CKD, chronic kidney disease; HR, heart rate; HRV, heart rate variability; HTN, hypertension; SNS, sympathetic nervous system; T2DM, type 2 diabetes mellitus. Created with BioRender.com.

Here, we suggest that monitoring non-dipping HR by consumer-grade wrist-worn devices may be of help in cardiovascular risk assessment in hypertension (Figure 1). Our suggestion is based on the following:

- Non-dipping HR is an independent, yet somewhat neglected, cardiovascular risk factor (1, 2, 5).
- Consumer-grade wrist-worn devices are able to continuously or almost continuously (HR measurements are taken every few minutes) monitor HR during a 24-h period (or even longer), encompassing daytime activities and night-time sleep. Moreover, the HR readings are reliable and accurate, with at least 90% agreement with 24-h ECG (35).
- Simultaneously with HR monitoring, wearable devices can track physical activity and sleep (36–38), thus allowing for better determination of bedtime (or sleep-time) HR decline. Indeed, in a randomized cross-over trial and validation study involving 32 healthy individuals, a wrist-worn device accurately determined sleep duration, dream sleep and slow wave sleep and had a very low bias and precision error for sleep-time HR and respiratory rate measurements when compared with ECG and polysomnography (39). Proper determination of the sleep/wake cycle and sleep-time HR is essential for non-dipping HR monitoring.
- Compared to conventional continuous cardiac monitors, 98% of surveyed patients deemed wrist-worn devices more convenient and 91% acknowledged they were likely to use a wrist-worn device to determine cardiac rhythm during symptoms (40). Indeed, using the patient's own wearable device can eliminate the anxiety associated with using assigned conventional cardiac monitors, thus improving measurement validity. Moreover, it is of no financial burden to the healthcare provider.
- Data collected by wearable devices can be automatically transmitted to smartphones for patient notification and stored in remote servers (cloud) or electronic health records to be accessed remotely by a healthcare professional. This ensures timely data collection and the avoidance of errors by subjective data reporting or secondary data entry, which may contribute to increased patient convenience, satisfaction and compliance, as well as reduced in-office visits, administrative burden and healthcare costs (15, 41, 42).
- The COVID-19 pandemic brought about new challenges in delivering health care to patients with chronic illnesses, including hypertension and diabetes mellitus (43). With social distancing and shelter-in-place, outpatient care is generally provided remotely *via* telemedicine. The proper management of chronic illnesses is also imperative under these circumstances (44). Monitoring non-dipping HR with wearable devices can provide valuable information on cardiovascular risk in patients with chronic illnesses even during lockdowns and social distancing measures.
- Importantly, HR monitoring with wearable devices could play a pivotal role in clinical research pertaining to sleep-time HR or non-dipping HR in various cardiovascular pathologies. Indeed, wearable devices can continuously and during any time interval collect data from large populations encompassing thousands of healthy subjects or subjects with hypertension or diabetes mellitus and associated cardiovascular affliction, such as ischemic heart disease, heart failure or stroke. In fact, large populations can be stratified into specific subpopulations by age, gender, demographics, and other factors, thus allowing for larger and denser datasets and advanced analyses (41). Population-based data on non-dipping HR (or rising HR) at bedtime may not only improve the pathophysiological understanding of this phenomenon but also show its prognostic value in cardiovascular and even other pathologies, thus rendering non-dipping HR a risk factor and marker of prognosis. Finally, data gathered by wearable devices could help to develop a well-tailored therapeutic approach targeting non-dipping HR (1, 3, 45, 46).

## Conclusion

Considering the above remarks, monitoring non-dipping HR by consumer-grade wrist-worn devices can be construed as an emerging avenue for cardiovascular risk assessment in hypertension, particularly in the realm of primary care. As primary care often struggles with high healthcare costs and a shortage of physicians or cutting-edge diagnostic tools, the suggested cost-effective approach to cardiovascular risk assessment using wearable devices may be advantageous. Moreover, early identification and management of high-risk patients in primary care can curb long-term healthcare expenditures (42, 47).

## Author Contributions

TB conceived and drafted the manuscript. FS revised the manuscript. All authors participated in data analysis, interpretation, and approved the submitted version.

## Conflict of Interest

The authors declare that the research was conducted in the absence of any commercial or financial relationships that could be construed as a potential conflict of interest.

## Publisher's Note

All claims expressed in this article are solely those of the authors and do not necessarily represent those of their affiliated organizations, or those of the publisher, the editors and the reviewers. Any product that may be evaluated in this article, or claim that may be made by its manufacturer, is not guaranteed or endorsed by the publisher.
